# CRP and TNF-α  Induce PAPP-A Expression in Human Peripheral Blood Mononuclear Cells

**DOI:** 10.1155/2012/697832

**Published:** 2012-09-11

**Authors:** Weiping Li, Hongwei Li, Fusheng Gu

**Affiliations:** Department of Cardiology, Beijing Friendship Hospital Affiliated to Capital Medical University, Beijing 100050, China

## Abstract

*Objective*. The effects of C-reactive protein (CRP) and tumor necrosis factor-**α** (TNF-**α**) on pregnancy-associated plasma protein-A (PAPP-A) expression in human peripheral blood mononuclear cells (PBMCs) require further investigation. *Methods*. The PAPP-A levels in culture supernatants, PAPP-A mRNA expression, and cellular PAPP-A expression were measured in human PBMCs isolated from fresh blood donations provided by 6 healthy volunteers (4 donations per volunteer). Analyses were conducted by ultrasensitive ELISA, western blotting, and RT-PCR following stimulation with CRP or TNF-**α** cytokines. *Results*. PAPP-A mRNA and protein levels after CRP stimulation peaked at 24 hours, whereas peak PAPP-A mRNA and protein levels were achieved after TNF-**α** stimulation at only 2 and 8 hours, respectively. These findings indicate the dose-dependent effect of CRP and TNF-**α** stimulation. Actinomycin D treatment completely prevented CRP and TNF-**α** induction of PAPP-A mRNA and protein expression. Additionally, nuclear factor- (NF-) **κ**B inhibitor (BAY11-7082) potently inhibited both CRP and TNF-**α** stimulated PAPP-A mRNA and protein expression. *Conclusions*. Human PBMCs are capable of expressing PAPP-A *in vitro*, expression that may be regulated by CRP and TNF-**α** through the NF-**κ**B pathway. This mechanism may play a significant role in the observed increase of serum PAPP-A levels in acute coronary syndrome (ACS).

## 1. Introduction

Pregnancy-associated plasma protein-A (PAPP-A) is a metzincin metalloproteinase primarily produced by the placental syncytiotrophoblast during pregnancy. PAPP-A is also synthesized by fibroblasts, osteoblasts, vascular smooth muscle cells (VSMCs), and endothelial cells (ECs). *In vitro*, PAPP-A functions to cleave insulin-like growth factor-binding protein 4 (IGFBP-4), an inhibitory IGFBP, consequently increasing IGF bioavailability for receptor activation [1–4]. *In vivo*, several studies have shown a similar role for PAPP-A in modulating site- and event-specific IGF signaling during injury repair processes [2].

Recent studies have indicated that PAPP-A is a novel biomarker for plaque instability and inflammation useful in early diagnosis, risk stratification, and prognostic prediction in patients with acute coronary syndrome (ACS) [5, 6]. PAPP-A was found abundantly expressed in ruptured and eroded human atherosclerotic plaques, colocalized with activated smooth muscle cells and macrophages [7, 8]. Since plaque-derived PAPP-A is being considered as a new biomarker that may potentially play a role in the development of atherosclerotic lesions [9, 10]. A better understanding of its cellular source and regulation is important to the future development and implementation of therapeutics utilizing this biomarker. 

Previous studies have indicated that proinflammatory cytokines, interleukin- (IL-) 1*β*, and tumor necrosis factor- (TNF-) *α* were potent stimulators of PAPP-A expression in cultured human fibroblasts, osteoblasts, coronary artery smooth muscle cells, and endothelial cells (ECs) [9, 11]. Despite these significant findings, little is known about the effect of C-reactive protein (CRP) and TNF-*α* on PAPP-A expression in human peripheral blood monocytes (PBMCs).

The current study investigates the ability of CRP and TNF-*α* to induce PAPP-A expression in the PBMCs of healthy volunteers. Furthermore, inhibitor experiments have been designed to explore the underlying intracellular signaling pathways involved in PAPP-A expression. The focus of these studies is nuclear factor- (NF-) *κ*B pathways, a major pathway associated with cytokine stimulation in various cell types.

## 2. Material and Methods

### 2.1. Study Participants and Sampling

Peripheral blood was collected from the forearm vein of healthy volunteers enrolled in the current study. For each experiment, 30 mL samples were freshly collected from 6 healthy subjects for use in PBMC preparations. Each of these 6 subjects provided 4 blood donations over the course of the study. The study was approved by the Ethics Committee of the Beijing Friendship Hospital and conforms to the principles outlined in the Declaration of Helsinki. All participants provided written informed consent prior to enrollment.

### 2.2. PBMC Separation and Culture

Human PBMCs were isolated from 30 mL fresh blood samples obtained from healthy volunteers by Ficoll-Paque (Amersham Bioscience, Uppsala, Sweden) centrifugation. Resultant cells were washed 3 times with PBS and subsequently resuspended in RPMI 1640 (GibcoBRL, Grand Island, NY, USA) supplemented with 10% fetal calf serum (GibcoBRL, Grand Island, NY, USA), penicillin (100 U/mL), and streptomycin (100 *μ*g/mL). Cells were then cultured for 24 hours in plastic dishes at 37°C in a humidified atmosphere of 5% CO_2_. Upon observation of subconfluent growth, the medium was replaced with fresh medium. All nonadherent lymphocytes were discarded during the medium change, reserving only healthy adherent monocytes. After 24 hours, trypan blue exclusion indicated that 95% of cultured PBMCs were living. PBMCs were plated at a final concentration of 1 × 10^6^ cells/mL for all stimulation and pharmacological studies. 

**Figure 1:**
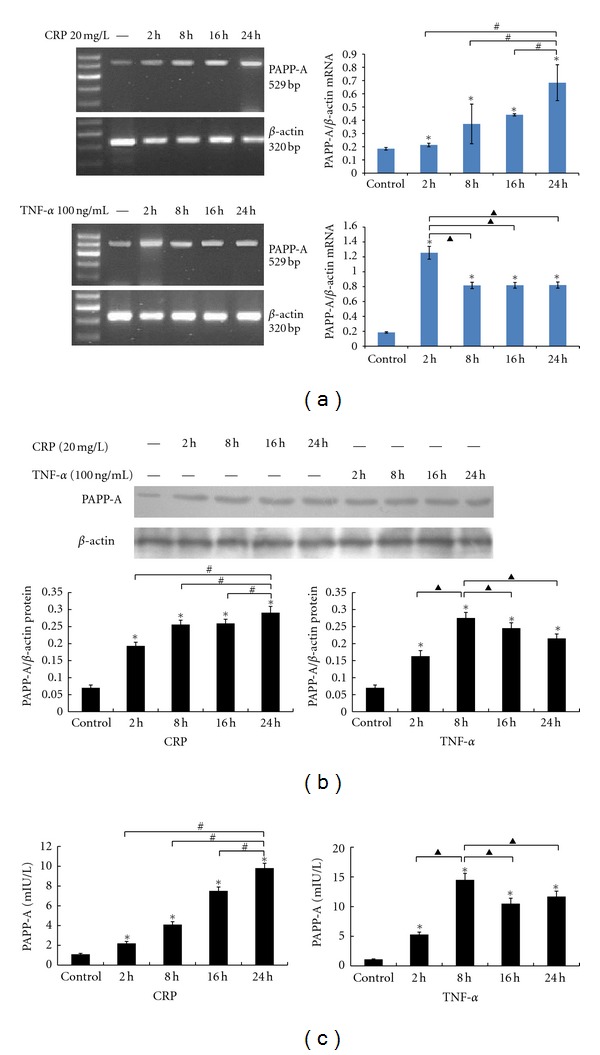
PBMCs were stimulated with CRP or TNF-*α* for 2, 8, 16, 24 hours. (a) PAPP-A mRNA were measured using RT-PCR. (b) PAPP-A protein expression were measured by western blotting. (c) Culture supernatant was collected respectively to analyze PAPP-A concentration by an ultra-sensitive ELISA. The resultant data are expressed as the means ± SD of six donors. ∗: *P* < 0.05 compared with the control. #: *P* < 0.05 compared with the PBMCs stimulated with CRP for 24 hours; ▲: *P* < 0.05 compared with the PBMCs stimulated withTNF-*α* for 2 hours or 8 hours.

**Figure 2:**
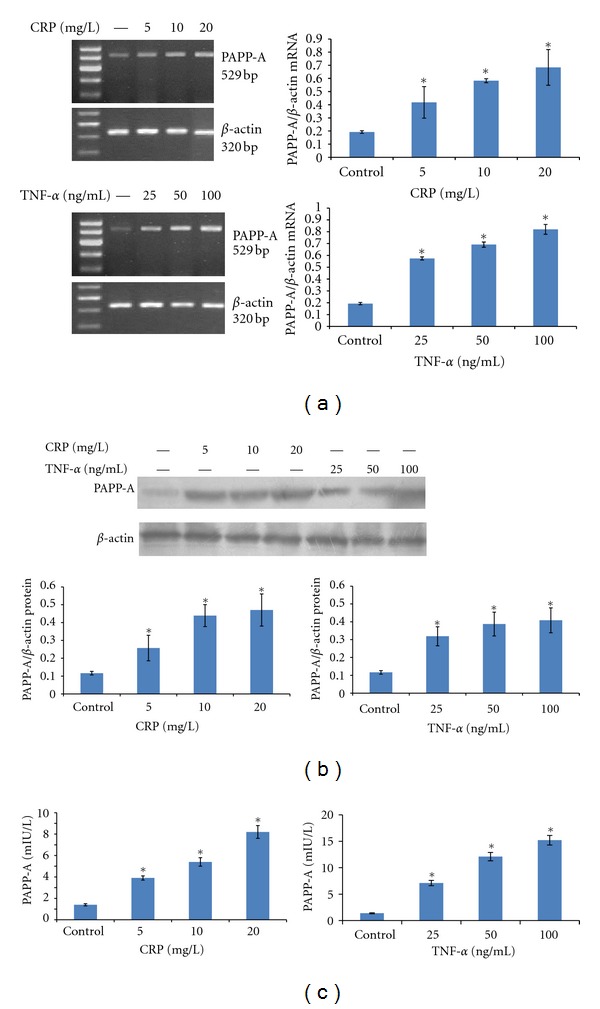
PBMCs were stimulated with CRP (5, 10, or 20 mg/L) or TNF-*α* (25, 50, or 100 ng/mL) for 24 hours. (a) PAPP-A mRNA were measured using RT-PCR. (b) PAPP-A protein levels were measured by Western blotting. (c) Culture media was collected for analysis of PAPP-A concentrations by ultrasensitive ELISA. The resultant data are expressed as the means ± SD of six donors. ∗: *P* < 0.05 compared with the control.

**Figure 3:**
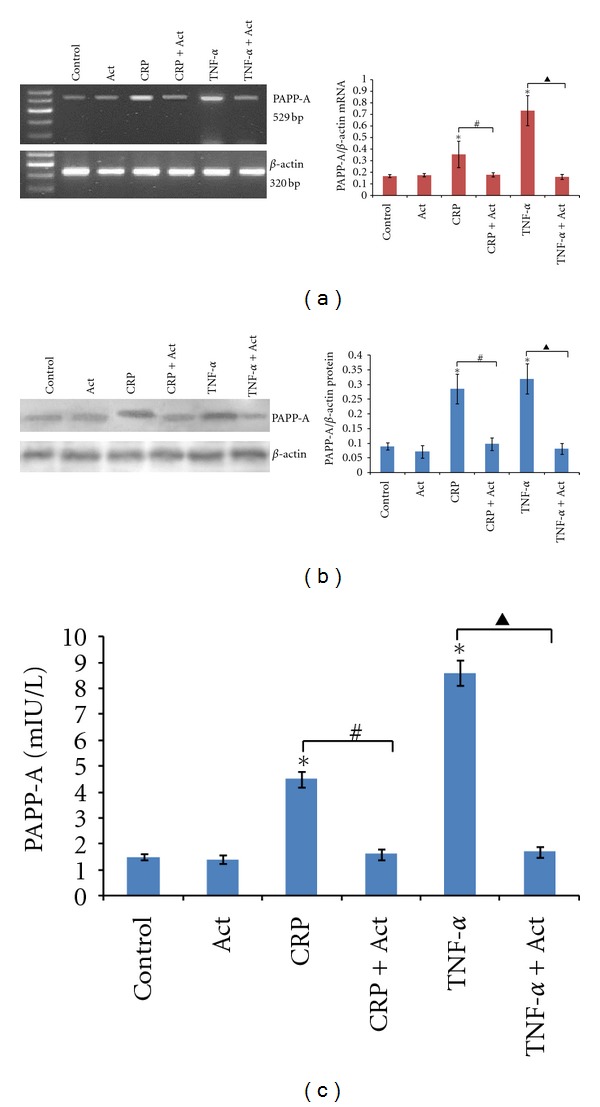
Regulation of PAPP-A expression: effects of actinomycin D. PBMCs were treated with or without (control) 20 mg/L CRP or 100 ng/mL TNF-*α* in the absence or presence of actinomycin D (1 *μ*g/mL). RT-PCR was performed on RNA, (a) Western blotting was performed on cell lysates, (b) and an ultrasensitive ELISA was performed on conditioned medium (c) after 8 h treatment. The results are expressed as the means ± SD of six donors. ∗: *P* < 0.05 compared with the control; #: *P* < 0.05 the CRP group versus the CRP + Act group; ▲: *P* < 0.05 the TNF-*α* group versus the TNF-*α* + Act group.

**Figure 4:**
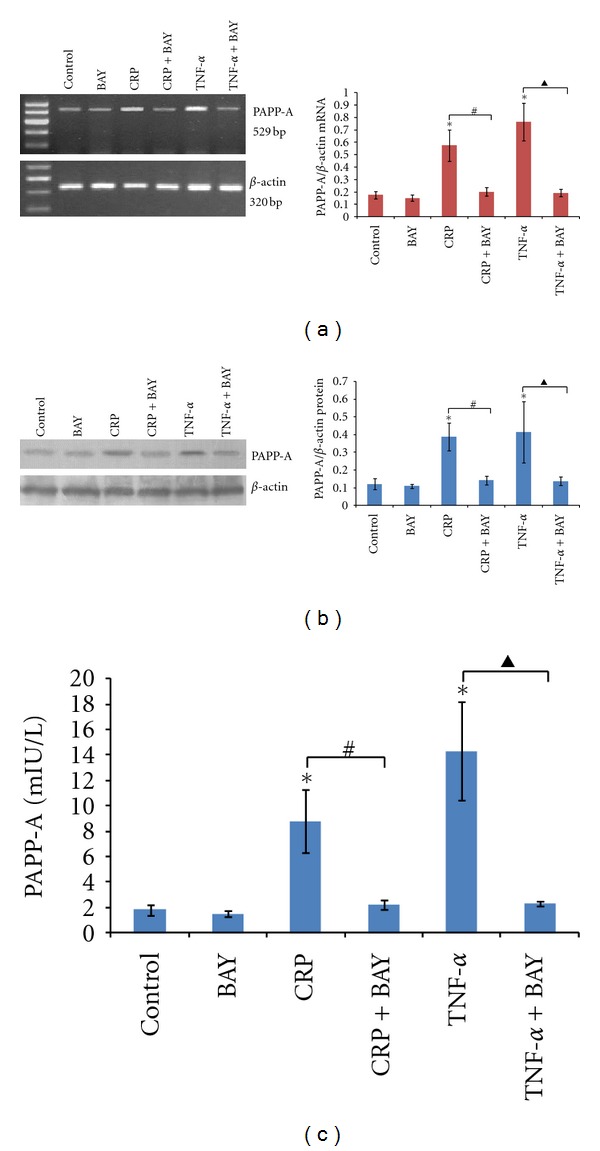
Effect of BAY11-7082 on cytokine-induced PAPP-A expression. PBMCs were pretreated with BAY11-7082 (20 *μ*M) for 60 min and then treated with CRP (20 mg/L) or TNF-*α* (100 ng/mL) for 24 hours. (a) Detection of PAPP-A mRNA expression was conducted by RT-PCR. (b) PAPP-A protein expression were determined by Western blotting. (c) PAPP-A concentrations were performed by an ultra-sensitive ELISA. The results are expressed as the means ± SD of six donors. ∗: *P* < 0.05 compared with the control; #: *P* < 0.05 the CRP group versus the CRP + BAY group; ▲: *P* < 0.05 the TNF-*α* group versus the TNF-*α* + BAY group.

The pharmacological agents recombinant human TNF-*α* (Perprotech, Rocky Hill, CT, USA), CRP, actinomycin D, and BAY11-7082 (Sigma Chemicals, Deisenhofen, Germany) were dissolved into solution according to the manufacturer's instructions. Resultant solutions were added to cultured PMBCs at defined time intervals (2, 8, 16, 24 hours) and concentrations (CRP: 5, 10, or 20 mg/L; TNF-*α*: 25, 50, or 100 ng/mL; BAY11-7082: 20 *μ*M) in the absence or presence of actinomycin D (1 *μ*g/mL), as further described in the following sections. Individual controls were determined for each experiment, as described in Figures 1–4. 

### 2.3. Semiquantitative Reverse Transcription Polymerase Chain Reaction (RT-PCR) Detection of PAPP-A mRNA

Briefly, total RNA was isolated using TRIzol (Invitrogen, Calsbad, CA, USA) according to the manufacturer's instructions. Reverse transcription-generating cDNA was performed using the SuperScript III First-Strand Synthesis System (Invitrogen, Calsbad, CA, USA). PAPP-A cDNA was amplified using forward (5′-ATA TCT CAC GTG ACC GAG GA-3′) and reverse (5′-AGA TGA TGG TGC TGG AAG TC-3′) primers, which produce a 529 bp product. Amplification was performed at 94°C for 2 min for preheating, followed by 30 cycles of 94°C for 45 s, 65°C for 45 s, 72°C for 60 s, and a final extension of 72°C for 10 min. *β*-actin was amplified using forward (5′-GCA TGG AGT CCT GTG GCA T-3′) and reverse (5′-CTA GAA GCA TTT GCG GTG G-3′) primers, which produce a 320 bp product. Amplification was performed at 94°C for 2 min for preheating, followed by 28 cycles of 94°C for 30 s, 60°C for 30 s, 72°C for 30 s, and a final extension of 72°C for 20 min. 

PCR products were electrophoresed on 1.5% agarose gels containing ethidium bromide. The resulting bands were photographed under ultraviolet light and analyzed using a Gel Imaging System (Gel Doc2000, Bio-Rad, Hercules, CA, USA). The relative intensity of bands of interest was expressed as the ratio to *β*-actin mRNA bands.

### 2.4. Western Blot Analysis of PAPP-A Protein Expression

For cell lysates, cells were washed twice with ice-cold PBS and lysed in RIPA buffer. Total protein was quantified using the BCA assay (Pierce, Rockford, IL, USA). Equal amounts of protein (40 *μ*g) were separated by SDS-PAGE in 14% Tris-glycine gels (TEFCO, Tokyo, Japan). After electrophoresis, the proteins were blotted onto a nitrocellulose membrane and blocked with 5% skim milk powder diluted in Tris-buffered saline (TBS) with 0.05% Tween 20. Rabbit polyclonal antibodies against human PAPP-A (1 : 1000, Abcam Systems, Cambridge, USA) were used as the primary antibody. Membranes were incubated with diluted antibody preparations overnight at 4°C. After washing the next day, membranes were incubated with horseradish peroxidase- (HRP-) conjugated affinity-purified goat anti-rabbit IgG antibody (1 : 3000, Santa Cruz. Biochemical, California, USA) for 1 hour at room temperature. The blots were visualized using enhanced chemiluminescence reagents and autoradiography. The PAPP-A quantitative relative expression was calculated by comparison with *β*-actin.

### 2.5. Analysis of PAPP-A Level in Culture Supernatants by ELISA

Culture supernatants were collected and stored at −80°C. PAPP-A levels were determined using the ultrasensitive ELISA kit (Diagnostic Systems Laboratories, Webster, TX). The assay was calibrated using recombinant PAPP-A calibrated against the World Health Organization's international reference preparation 78/610 for pregnancy-associated proteins, by definition containing a PAPP-A concentration of 100 IU/L. Minimum sensitivity was 0.24 mIU/L with intra- and interassay coefficients of variation of 4.7% and 4.2%, respectively.

### 2.6. Statistical Analysis

All statistical analyses were carried out using the SPSS statistical package, version 13.0 (SPSS Inc., Chicago, IL, USA) for Windows. Results were expressed as the means ± SD of six donors. ANOVA was used for comparisons between multiple groups. *P*-values less than 0.05 were considered statistically significant (*P* < 0.05). 

## 3. Results

### 3.1. CRP and TNF-*α* Induce PAPP-A Expression in PBMCs and Protein Level in Culture Supernatants

The time course of PAPP-A mRNA expression in PBMCs under basal and cytokine-stimulated conditions is presented in Figure 1(a). Little PAPP-A expression was observed in PBMC cultures under basal conditions after 24 hours. Treatment with CRP (20 mg/L) or TNF-*α* (100 ng/mL) significantly increased PAAP-A mRNA expression at all time points (2, 8, 16, 24 hours). PAPP-A mRNA levels increased 2 hours after stimulation with CRP (20 mg/L) and remained elevated by approximately 3.7-fold up to 24 hours. PAPPP-A mRNA expression, however, rapidly increased and peaked at approximately 6.8-fold 2 hours after TNF-*α* (100 ng/mL) stimulation. A subsequent decrease was then observed, though levels remained elevated at approximately 4.5-fold up to 24 hours. Maximal PAPP-A protein expression in PBMCs and concentrations in culture supernatants were achieved with CRP stimulation by 24 hours and TNF-*α* stimulation by 8 hours (Figures 1(b) and 1(c)), reflecting the changes in PAPP-A mRNA expression.

As shown in Figure 2, dose-response experiments confirmed CRP or TNF-*α* treatment elicited dose-dependent increases in PAPP-A mRNA expression, protein expression in PBMCs, and secretion in the supernatant after 24 hours. CRP showed half-maximal effectiveness at approximately 5 mg/L, with maximal effectiveness at approximately 20 mg/L (Figure 2). TNF-*α* showed half-maximal effectiveness at approximately 25 ng/mL, with maximal effectiveness at approximately 100 ng/mL.

### 3.2. mRNA Synthesis Dependence of CRP or TNF-*α* on PAPP-A Expression in PBMCs

The dependence of PAPP-A expression on mRNA synthesis was explored in the following three experiments.  Figure 3 showed that the effects of these proinflammatory cytokines appeared to be at the level of transcription, as the DNA-directed RNA polymerase inhibitor, actinomycin D, completely prevented CRP or TNF-*α* induction of PAPP-A mRNA expression, protein expression, and concentrations in culture supernatants. These results showed that CRP or TNF-*α* was responsible for new protein synthesis of the PAPP-A protein. Furthermore, PAPP-A protein was actively secreted into the supernatant.

### 3.3. CRP and TNF-*α* Induced PAPP-A Expression via NF*κ*B

As indicated in our previous experiments, treatment of human PBMCs with CRP (20 mg/L) or TNF-*α* (100 ng/mL) significantly increased PAPP-A mRNA expression, protein expression, and concentrations in culture supernatants. To confirm the role of NF*κ*B activation, BAY11-7082, which inhibits inducible phosphorylation of I*κ*B, was shown to effectively inhibited CRP and TNF-*α*-stimulated PAPP-A expression (Figures 4(a), 4(b), and 4(c)). It is well known that the NF*κ*B pathway is the critical mediator of prooxidant stimuli, such as inflammatory cytokines [12]. The basic mechanism by which NF*κ*B is activated is through phosphorylation of intrinsic inhibitors, with the I*κ*B, subsequently freeing NF*κ*B to translocate into the nucleus where it regulates gene expression.

## 4. Discussion 

Novel markers of coronary artery disease progression have been confirmed in recent years, with circulating levels of PAPP-A standing out as one of the most prominent indicators of this profile. The current study indicates that PAPP-A expression in human PBMCs may be regulated by CRP and TNF-*α* through the NF-*κ*B pathway, a mechanism that may play a critical role in increases in serum PAPP-A levels during acute coronary syndrome (ACS). These findings are consistent with previous reports indicating that PAPP-A is a marker of atheromatous plaque instability as well as extent and prognosis of cardiovascular disease [13–15]. Serum PAPP-A levels increase in patients with ACS, indicating that PAPP-A may also be a marker of adverse events [16–18]. Moreover, in chronic stable angina (CSA) patients, PAPP-A is an independent predictor for the extent of vessel stenosis, where it has been shown to correlate with the presence of vulnerable coronary artery stenosis [19, 20]. Thus, PAPP-A levels have demonstrated a firm association with angiographic plaque complexity in CSA patients [21].

The stimulation of PAPP-A expression by TNF-*α* has been observed previously in human fibroblasts, osteoblasts, VSMCs, and ECs [4, 11, 22]. Using specific monoclonal antibodies, Bayes-Genis et al. reported that PAPP-A was abundantly expressed in both eroded and ruptured plaques, but was only minimally expressed in stable plaques [7]. Moreover, in plaques with large lipid cores and cap rupture, staining for PAPP-A occurred mostly in the inflammatory shoulder region, in areas surrounding the lipid core, and in areas with localized CD68-positive cells. Thus, PAPP-A levels have been associated with inflammation in regions of atherosclerotic plaques, potentially contributing to progression and poor outcomes in patients. 

Most evidence of monocyte involvement of PAPP-A expression has been completed through analysis of atherosclerotic plaques, where monocytes are the predominant leukocyte contributing to the development, progression, and instability of atherosclerotic lesions, they often contain high levels of PAPP-A [23]. Because samples of plaques may contain a mixture of leukocytes and circulating compounds, these tests have left the source of PAPP-A observed in these monocytes a point of debate among researchers. Double immunofluorescence confocal microscopy (ICM) has been used to characterize cell types expressing PAPP-A, suggesting that monocytes are the primary source of PAPP-A in plaques and the target cells for cytokines [24]. It has also been speculated that PAPP-A may be produced by activated monocytes or macrophage cells in unstable plaques and released into the extracellular matrix and circulation [10]. Conversely, Conover et al. suggested that *in vivo* macrophages actually failed to produce PAPP-A, but instead internalized the compound through their membranes, thus accounting for the accumulation of circulating PAPP-A produced by other sources in macrophage cells associated with plaques [25]. The current study, however, indicates that the specific mRNA expression associated with PAPP-A production increases in macrophages *in vitro*, resulting in expression and supernatant secretion of PAPP-A. These findings indicate that PAPP-A production occurs in macrophages rather than being internalized through the membrane, as suggested by Conover et al. Further *in vivo* studies will be required to assess the affect of circulating PAPP-A levels on macrophage PAPP-A expression and secretion, which may account for the discrepancies between these two studies. Cumulatively, the findings of the current study indicate that macrophages in cultured human PBMCs can synthesize and secrete PAPP-A. 

Furthermore, CRP and TNF-*α* were indicated to be potent stimulators of PAPP-A gene expression and protein secretion in human PBMCs, suggesting a link between the increase in local inflammatory cytokine production and PAPP-A during ACS. While further studies of the *in vivo* effects in ACS patients will be required to confirm these results, these findings suggest that serum PAPP-A, hsCRP, and TNF-*α* levels may be significantly higher in ACS patients than in patients with stable angina pectoris and that increasing PAPP-A mRNA levels in patients with ACS may also have a positive association with serum hsCRP and TNF-*α* mRNA expression. Exploration of these effects is a topic being explored in our current research based on the initial positive findings of the current research. Furthermore, the time course of stimulation by CRP and TNF-*α* differed, with PAPP-A expression increasing 2 hours after stimulation with CRP, peaking at approximately 3.7-fold by 24 hours, whereas a rapid increase to approximately 6.8-fold was seen in only 2 hours with TNF-*α* stimulation. The current study provides significant evidence for PAPP-A production in PMBCs and stimulation by the cytokines CRP and TNF-*α*; however, further *in vivo* studies will be required to verify these findings and assess the effects of circulating PAPP-A on PMBC PAPP-A production.

Actinomycin D was observed to complete block the induction of PAPP-A mRNA expression by CRP and TNF-*α*, indicating potential regulation at the level of transcription. The rapid increase in PAPP-A mRNA levels after treatment with these cytokines further confirms the hypothesis of transcriptional regulation. As expected, increases in PAPP-A protein expression and secretion into the supernatants were paralleled by increases in gene expression. Based on this observation, it is possible that the biological consequence of CRP- and TNF-*α*-induced PAPP-A expression in human PBMCs was enhanced IGF-I bioactivity mediated by PAPP-A proteolysis of IGFBP-4, thus contributing to the progression of both coronary atherosclerosis and restenosis. It is well known that the free fraction of circulating and locally synthesized IGF-I stimulates VSMC proliferation, migration, and extracellular matrix synthesis. In macrophages, IGF-I also promotes excess LDL cholesterol uptake, production of proinflammatory cytokines, and chemotaxis [26, 27]. This inflammatory environment digests the fibrous cap and leaves the plaque prone to rupture. IGF-I also stimulates endothelial cell migration and organization, forming vascular conduits.

CRP and TNF-*α* were also identified as potential regulators of PAPP-A expression, operating through a mechanism involving the activation of the NF*κ*B system in PBMCs of healthy volunteers. NF*κ*B is a ubiquitous transcription factor that is activated by inflammatory cytokines, infection, oxidative stress, and shear stress. It is known for its key role in the inflammatory process [28–30]. Ritchie reported that NF*κ*B was activated in peripheral monocytes using an electromobility shift assay in patients suffering from unstable angina [31]. The NF*κ*B family of transcription factors plays a critical role in coordinating and regulating the expression of a wide variety of inflammatory genes that have been linked to the pathologies of ACS [32]. Some studies have even demonstrated that CRP induces NF*κ*B activity in various cell types, including peripheral monocytes, saphenous vein endothelial cells, and human aortic endothelial cells [23, 33, 34]. Moreover, Resch et al. reported that TNF-*α* induced I*κ*B*α* degradation and PAPP-A-regulated expression in human fibroblasts by TNF-*α* was mediated by NF*κ*B activation [9]. The current study also demonstrated that BAY11-7082 was a potent inhibitor of both CRP- and TNF-*α*-stimulated PAPP-A expression in human PBMCs. The view of NF*κ*B as a transcription factor for PAPP-A gene expression, however, will require further study to identify the promoter region and validate these findings.

The current study provides a variety of evidence to support the expression on PAPP-A by leukocytes in PMBCs, including increased PAPP-A-associated mRNA expression, PAPP-A expression, and PAPP-A secretion into the supernatant of fresh *in vitro* samples collected from healthy subjects. Furthermore, the cytokines CRP and TNF-*α* were shown to stimulate PAPP-A expression in these cells. Based on these findings and the previously observed association between PMBCs and ACS, it is likely that activated monocytes or macrophages in PMBCs surrounding developing artherosclerotic plaques may secrete proinflammatory cytokines, thus stimulating the expression and secretion of PAPP-A. Further studies will, however, be required to assess the effects of elevated PAPP-A concentrations on autocrine and paracrine mechanisms for the exacerbation of atherosclerosis procession and plaque rupture through IGFBP-4 cleavage and enhanced local IGF-I bioavailability. The current results, however, provide the fundamental mechanistic groundwork for further understanding of the entire mechanism associated with cytokine regulation of PAPP-A expression and IGF bioavailability. This understanding may lead to the future development of novel therapeutic targets for the treatment of ACS.
